# Impact of climate change and air pollution on childhood respiratory health

**DOI:** 10.1016/j.jped.2024.11.007

**Published:** 2025-01-31

**Authors:** Marilyn Urrutia-Pereira, Dirceu Solé

**Affiliations:** a Universidade Federal do Pampa (UNIPAMPA), Faculdade de Medicina, Uruguaiana, RS, Brazil; b Universidade Federal de São Paulo, Escola Paulista de Medicina, Departamento de Pediatria, Disciplina de Alergia, Imunologia Clínica e Reumatologia, São Paulo, SP, Brazil

**Keywords:** Air pollutants, Climate change, Human health, Exposoma, Children

## Abstract

**Objective:**

To assess the impact of climate change and air pollution on children's respiratory health.

**Data source:**

Narrative review of articles published in English, Portuguese, French, and Spanish in the last decade in the following databases: PubMed, Google Scholar, EMBASE, and SciELO. The keywords used in this search were: climate changes OR air pollution OR indoor pollutants OR wildfires AND human health OR children OR exposome.

**Data synthesis:**

Increases in extreme weather events, such as heat waves, forest fires, floods, droughts, hurricanes, and dust storms, put children's respiratory system health at greater risk.

**Conclusions:**

The growing global increase in respiratory diseases in recent decades raises questions about the impact of environmental factors resulting from industrialization, urbanization, and climate change on the individual's exposome. Understanding it better is a key point for better treatment.

## Introduction

The growing global increase in the prevalence of respiratory diseases in recent decades raises questions about the impact of environmental factors resulting from industrialization, urbanization, and climate change on the individual's exposome.^1^

The exposome is the total set of exposures to which an individual is submitted over time. This includes non-genetic factors, such as pollution, excessive sun exposure, allergens, microbiome, among others, and also how these exposures influence human development and health.^2^

The exposome consists of two domains, although there is considerable overlapping of both: (1) general, and specific external exposome, and (2) internal exposome. The general external exposome refers to social and economic factors, the urban or rural environment (where the person lives), and climatic factors. The specific external exposome refers to the immediate local environment and includes exposure to chemicals, diet, physical activity, tobacco, and infections. The internal exposome refers to the biological processes within the organism that involve molecules, internal chemical components, and biological reactions to external exposures and the internal microbiome.^3^

The comprehensive identification of environmental factors affecting pediatric health and well-being requires the understanding of the exposome, particularly during sensitive and critical developmental stages of life, as it provides invaluable help in creating effective prevention strategies.^2^

Hopkinson et al. draw attention to the concept of "**GETomics**", which encompasses potentially different, cumulative, and interactive genetic (G) and environmental (E) interactions that act over time (T) to influence epigenetic changes and/or immune responses and, ultimately, culminate in the development of health and/or disease.^4^

Childhood is a time of both rapid somatic growth and physiological development, during which several biological systems and organs are at different stages of maturation. Moreover, children suffer more and earlier from higher levels of exposure and are particularly susceptible to the respiratory impacts of climate change due to the combination of environmental physiological factors and the lack of health equity.^5^

The developing child's body, as well as their immune system, increases the potential chance of developing allergic diseases and infections since children have higher respiratory rates and greater exposure to toxins, proportionally, per kilogram of body weight and per unit of time. This susceptibility is further aggravated by frequent outdoor activities, which expose them to a range of environmental hazards, including extreme temperatures, air pollutants, and allergens.^5^

Antenatal or early-life adverse exposures increase the lifetime risk of pulmonary disease. These influences manifest themselves in three ways. The first occurs through processes that prevent individuals from reaching their maximum potential in terms of lung development and growth.^4^ The second is represented by processes that prepare the lungs to be more sensitive to subsequent insults, which contributes to the third: early and then continuous lung damage caused by exposure to inhaled toxic materials, including tobacco smoke, environmental and household pollution, and infections, as well as other stressors.^4^

These insults are particularly prevalent in low- and middle-income countries, often exacerbated by social deprivation, poverty and climate change.^6^

As the burning of fossil fuels continues to drive the global economy, the speed of climate change is accelerating, causing severe respiratory health impacts and large disparities regarding the degree of human suffering.^7^

Climate change has significant consequences for children's respiratory health, with the main contributing factors being temperature, humidity, air pollution, and extreme weather events. Increases in extreme weather events, such as heat waves, wildfires, floods, droughts, hurricanes, and dust storms, put children's respiratory system health at greater risk.^8^^,^^9^

## Epithelial barrier/immune system alterations

Natural disasters, which are increasingly frequent, can synergistically damage the physical integrity and functional efficacy of the epithelial barrier due to exposure to a wide range of stimuli, including antigens, allergens, heat stress, pollutants, and microbiota alterations.^10^

A broken epithelial barrier induces pro-inflammatory activation of epithelial cells and the production of alarmins, which stimulate the innate immune system and influence adaptive immunity, especially in terms of developing and preserving immune tolerance.^11^ The loss or failure of immune tolerance can instigate a wide spectrum of non-communicable diseases, such as autoimmune conditions, allergies, and respiratory diseases.^10^

These changes are associated with microbial dysbiosis, with a predominance of colonizing opportunistic pathogens and a decrease in commensals, with a consequent impact on the composition of the intestinal and airway microbiome, which may better explain the harm to human health.^12^

## Temperature

Extreme temperatures directly affect the airway epithelial barrier by facilitating the rupture of structural proteins (tight **junctions**) and by triggering inflammation, airway hyperreactivity, and thermoregulatory system impairment. As a consequence, there is an increase in tidal volume and respiratory rate, causing greater specific airway resistance and reflex bronchoconstriction, due to the activation of bronchopulmonary vagal C-fibers and upregulation of the transient receptor potential vanilloid (TRPV)1 and TRPV4. Heat shock proteins are also activated under heat stress and contribute to both epithelial barrier dysfunction and airway inflammation.^13^

Climate changes have led to a higher frequency of high environmental temperatures and higher rates of heat-related diseases, such as respiratory diseases. Compared to adults, children have behavioral and physiological differences that guarantee them additional vulnerability to heat.^14^

Higher concentrations of ozone and particulate matter have been documented at higher temperatures, which may explain the higher rate of exacerbation of chronic respiratory diseases and premature mortality.^7^ A positive and significant relationship between temperature, relative humidity, and allergic diseases was observed in children under five years of age, especially in girls.^15^

## Forest fires

The intensification of wildfires, mostly anthropogenic and criminal ones, has significant implications for planetary health and public health. Exposure to fine particulate matter (PM_2.5_) present in the smoke from these fires is linked to adverse health effects.^16^ Hotter, drier climates lead to longer and more intense wildfire seasons, harming air quality worldwide.^7^

Inhalation of PM_2.5_ from forest fires causes lung injury due to oxidative stress, local and systemic inflammation, airway epithelium damage, and increased vulnerability to infection.^16^

A recent systematic review showed that smoke inhalation from forest fires was associated with multiple adverse health outcomes for children and adolescents, with respiratory morbidities being the most significant, with a combined relative risk (RR) of 1.04 (95%CI: 0.96-1.12) for all-cause respiratory morbidity, RR = 1.11 (95%CI: 0.93-1.32) for asthma, and RR = 1.13 (95%CI: 1.05-1.23) for upper respiratory infection.^17^

According to Dhingra et al., exposure to wildfire smoke during the early periods of postnatal development affects subsequent respiratory health early in life, with earlier use of respiratory disease medications (1-12 weeks: hazard ratio (HR) = 1.094 per one-day increase in the weekly average of smoke days, 95%CI: 1.005-1.191; 13-24 weeks: HR = 1.108, 95%CI: 1.016.1.209.^18^

## Rainfall

Nassikas et al. demonstrated that exposure to higher short-term rainfall can trigger airway inflammation in adolescents, particularly among those with asthma. For each 2-mm increase in the 7-day moving average of precipitation, there was an increase in the fraction of exhaled nitric oxide (FeNO) by 4.0% (95%CI: 1.1-6.9). There was evidence of a change in the effect intensity according to asthma status: precipitation was associated with lower forced vital capacity (FVC) and higher FeNO among adolescents with asthma.^19^

## Outdoor pollution

Air pollution levels are expected to increase due to continued economic growth and population expansion in many areas of the world, and climate changes are expected to increase the frequency and intensity of extreme weather events, amplifying air pollution levels and worsening respiratory diseases.^20^

According to the State of Global Air – 2024 report, air pollution is the second risk factor for death and determined 8.1 million deaths worldwide in 2021, and it was associated with more than 700,000 deaths of children under five years old, being the second risk factor for death worldwide in this group, second only to malnutrition.^21^

Outdoor air pollution is ubiquitous, and no safe level of exposure has been identified for the most common air pollutants, such as ozone and particulate matter. Table 1 shows the levels of the main air pollutants recommended by the World Health Organization as maximum allowable levels: particulate matter (PM_2.5_ and PM_10_), ozone (O_3_), nitrogen dioxide (NO_2_), sulfur dioxide (SO_2_) and carbon monoxide (CO).^22^

**Table 1:** Air quality guidelines levels and interim targets for six key pollutants.^22^

Pollutant	Averaging time	Interim targets				AQG	Evidence certainty
		1	2	3	4		
PM_2.5_, μg/m^3^	Annual	35	25	15	10	5	High
	24-h^a^	75	50	37.5	25	15	High
PM_10_, μg/m^3^	Annual	70	50	30	20	15	High
	24-h^a^	150	100	75	50	45	High
O_3_, μg/m^3^	Peak season^b^	100	70	-	-	60	Low-moderate
	8-h^a^	160	120	-	-	100	High
NO_2_, μg/m^3^	Annual	40	30	20	-	10	Moderate-high
	24-h^a^	120	50	-	-	25	High
SO_2_, μg/m^3^	24-h^a^	125	50	-	-	40	Low-high
CO, μg/m^3^	24-h^a^	7	-	-	-	4	Moderate

a 99th percentile of the distribution of daily values (3-4 exceedance days per year).

b Average of daily maximum 8-h mean O_3_ concentration in a period of six consecutive months with the highest 6-month running average O_3_ concentation.

These levels have been frequently revised. Children are more susceptible to the damage caused by outdoor air pollution, which can cause and aggravate respiratory diseases,^23^ as they determine a higher risk of acute respiratory infections, asthma, and decreased lung function. This risk varies depending on the geographic region, the source of air pollution, and the duration and concentration of exposures.^24^

Exposure to NO_2_ was associated with reduced lung function and higher FeNO among generally healthy children and adolescents.^25^ Puvvula et al. identified positive and significant associations between the mean annual concentration of pollutants (PM_2.5_, CO, NO_2_, SO_2_) with race (non-Hispanic blacks and Hispanics/Latinos), financial stability, and literacy. There were significant and positive associations between higher rates of visits to the pediatric emergency department for asthma and neighborhoods with more non-Hispanic black children, children without health insurance coverage, and households without access to motor vehicles.^26^

PM_10_ and PM_2.5_ levels were also associated with a higher incidence of influenza-like illnesses, and NO_2_ concentrations were associated with a higher rate of children's hospitalizations due to respiratory syncytial virus infections of the lower respiratory tract.^27^

## Indoor pollution

The poor quality of housing affects the air quality inside it and significantly impacts the respiratory health of children and young people. Exposure to humidity and/or mold in the home, cold homes, and the presence of pests and pollutants have a significant detrimental impact on children's respiratory health.^28^

Respiratory infections, particularly in children, and other chronic respiratory diseases, are strongly attributable to household air pollution. The elimination of these exposures through interventions such as the use of cleaner fuels and, preferably, electricity, is essential to improve the respiratory health of these individuals.^29^

A recent report by the Lancet Countdown 2024 points out that globally, 30% of households still depend on biomass burning to meet their energy and food preparation needs, and PM_2.5_ indoors, due to the burning of domestic solid fuels, determined 2.3 million deaths in 65 countries in 2020.^30^

## Pollutants/Allergens

Children are particularly vulnerable to respiratory diseases caused and exacerbated by aeroallergens, pollutants, and infectious agents.^5^

Due to climate change, the atmospheric content of triggering factors such as pollen and fungi increases and induces rhinitis and asthma in sensitized patients eliciting IgE-mediated allergic reactions. Pollen allergens trigger the release of pro-inflammatory mediators and accelerate the onset of sensitization to other respiratory allergens in predisposed children and adults. Lightning storms during pollen seasons can further aggravate the intensity of respiratory allergy and asthma not only in adults but also in children with pollinosis.^31^

## Conclusion

The climate crisis is a major public health threat to children and disproportionately affects the most vulnerable populations. Climate changes cause a multitude of health problems for this age group, especially respiratory ones.^32^

Most of the available evidence suggests positive benefits for the respiratory health of children and adolescents resulting from greenhouse gas mitigation actions that simultaneously reduce air pollution (specifically PM_2.5_ and NO_2_).^33^

Caregivers and parents of children with respiratory problems have high levels of concern regarding climate change and report adverse impacts on their children's health, especially if they have asthma.^34^

Increased awareness and qualification among pediatricians are needed to better understand the impact of climate change on children's health and educate parents on preventive, mitigation, and adaptation measures, such as limiting outdoor activities during pollution peaks, which are essential to preserving children's respiratory health.

Obtaining a detailed pediatric environmental history ^35^ helps to identify risk factors. It helps to understand:

• the quality and extent of hazards in environments where the child stays or spends time;
• to identify suspicious patterns or aspects that require further evaluation; and
• to determine the association between environmental factors and symptom onset, worsening, and improvement.^36^

To address climate change issues, professional pediatric associations must increase their advocacy with government agencies and consider climate change as representing a pediatric health emergency.

## Conflicts of interest

The authors declare no conflicts of interest.
